# “I Can't Find Anything Wrong: It Must Be a Pulmonary Embolism”: Diagnosing Suspected Pulmonary Embolism in Primary Care, a Qualitative Study

**DOI:** 10.1371/journal.pone.0098112

**Published:** 2014-05-19

**Authors:** Marie Barais, Nathalie Morio, Amélie Cuzon Breton, Pierre Barraine, Amélie Calvez, Erik Stolper, Paul Van Royen, Claire Liétard

**Affiliations:** 1 Université de Bretagne Occidentale, Faculté de Médecine et des Sciences de la Santé de Brest, Département Universitaire de Médecine Générale, Brest, France; 2 Faculty of Health, Medicine and Life Sciences, Caphri School for Public Health and Primary Care, Department of Family Medicine, Maastricht University, Maastricht, The Netherlands; 3 Department of Primary and Interdisciplinary Care, University of Antwerp, Antwerp, Belgium; 4 Université Européenne de Bretagne, Faculté de Médecine et des Sciences de la Santé de Brest -Laboratoire de Santé Publique, Epidémiologie, Brest, France; Queensland Institute of Medical Research, Australia

## Abstract

**Background:**

Before using any prediction rule oriented towards pulmonary embolism (PE), family physicians (FPs) should have some suspicion of this diagnosis. The diagnostic reasoning process leading to the suspicion of PE is not well described in primary care.

**Objective:**

to explore the diagnostic reasoning of FPs when pulmonary embolism is suspected.

**Method:**

Semi-structured qualitative interviews with 28 FPs. The regional hospital supplied data of all their cases of pulmonary embolism from June to November 2011. The patient's FP was identified where he/she had been the physician who had sent the patient to the emergency unit. The first consecutive 14 FPs who agreed to participate made up the first group. A second group was chosen using a purposeful sampling method. The topic guide focused on the circumstances leading to the suspicion of PE. A thematic analysis was performed, by three researchers, using a grounded theory coding paradigm.

**Results:**

In the FPs' experience, the suspicion of pulmonary embolism arose out of four considerations: the absence of indicative clinical signs for diagnoses other than PE, a sudden change in the condition of the patient, a gut feeling that something was seriously wrong and an earlier failure to diagnose PE. The FPs interviewed did not use rules in their diagnostic process.

**Conclusion:**

This study illustrated the diagnostic role of gut feelings in the specific context of suspected pulmonary embolism in primary care. The FPs used the sense of alarm as a tool to prevent the diagnostic error of missing a PE. The diagnostic accuracy of gut feelings has yet to be evaluated.

## Introduction

Pulmonary Embolism (PE) is a serious pathology which has to be identified quickly: the mortality rate is high, with 18% of patients dying within 3 months [1].The incidence of PE ranged from 23 to 60 per 100 000 [2], [3]. PE was clinically suspected in fewer than half of all fatal cases [4]. Because of the low rate of autopsy, the actual incidence is likely to be higher [2].

Uncertainty is an inherent part of primary care [5], [6]. Signs and symptoms are often vague. Dyspnea and thoracic pain are signs indicating multiple pathologies from the benign to a life-threatening PE [7]. FPs have to select patients with a serious pathology in order to refer them to secondary care or an emergency unit. They are torn between missing a patient with a hypothetical PE and referring too many patients for harmful and costly investigations. Pulmonary embolism was one of the most frequently reported missed diagnoses in primary care [8]. However, only 10% of cases of suspected PE turned out to be actual pulmonary embolisms [9].

How to safely exclude a diagnosis of PE is now well described. A literature review asserted the efficiency of the Wells rule and a D-dimer test in excluding the diagnosis [10]. This diagnostic approach was also validated in primary care. A Wells score <4 combined with a negative point of care D-dimer test were proven safe and efficient for the exclusion of a PE diagnosis in primary care [11].

In fact before using any prediction rule oriented towards this particular diagnosis, the GP should have some suspicion of PE and it is precisely this initial stage which is unclear. The diagnostic process leading to the suspicion of PE is not well described in primary care. The objective of this study was therefore to explore how FPs came to suspect pulmonary embolism in real settings: how the suspicion of PE developed in the diagnostic reasoning process of French FPs. A second objective was to describe more specifically the role of gut feelings in this diagnostic process.

## Materials and Methods

A qualitative approach was chosen because this type of research would enable us to explore the meanings of diagnostic signs and symptoms used in the diagnostic reasoning process of FPs [12]. Individual face-to-face semi-structured interviews were carried out with FPs. Interviews were chosen because we wanted to access to the personal experience of each FP, and not that of the group of FPs as a whole. We aimed to reveal issues which had not been documented previously [13]. It was conducted within a grounded theory perspective in order to describe from the data the way FPs perform the diagnostic process [14].

### Ethics statement

This study was approved by the ethic committee of the University de Bretagne Occidentale. The participants provided their written informed consent to participate in this study. The ethic committee approved this consent procedure.

### Research team

The research team included two FPs (MB and PB) with academic backgrounds and two female trainees (NM and AC) in family practice doing their Master's degree. After being coached in interview techniques, the two trainees conducted all the interviews. The theme of pulmonary embolism was cited during the call to request an appointment.

### Participant selection

Two groups of participants were selected. The first group consisted of 14 FPs who had referred a patient to the emergency unit of the local hospital in the area of Brest, Brittany and where a pulmonary embolism was ultimately diagnosed. Data of all the cases of pulmonary embolism from June to November 2011 were collected. The patient's FP was identified where he/she had been the physician who had sent the patient to the emergency unit. We had no information on the reason why the physician referred the patient to the emergency unit when we interviewed him/her. We undertook the FPs' interviews a few days after the positive diagnosis of PE. The first consecutive 14 FPs who agreed to participate made up the first group. A second group of FPs was chosen using a purposeful sampling method. The aim of selecting a second group was to include the widest possible range of perspectives, experiences, points of view, and, in particular, to enrich the sample with FPs from rural areas who had not referred their patients to the regional hospital. Sixty FPs were approached through a phone call during which the theme of the interview was presented. Fourteen agreed to participate and they made up the second group. Reasons to decline included prior engagements and lack of time. Information on participant profiles is detailed in the “results” section.

### Data Collection

The research team developed the interview form with a topic guide, drawn up to answer the research question (see table 1). It was composed of open-ended questions for exploration and closed questions to refine the participants' answers. The first question focused on the case report of a hospitalized patient for the first group and a recollected consultation about a positive diagnosis of PE for the second group. In order to match what emerged from the interviews, we added new questions to the interview guide when participants raised aspects which had not previously been mentioned [15]. All the interviews were audio-recorded. The recordings were transcribed and checked. All the interviews took place at the FP's office. In order to improve validity and credibility, all transcripts were returned to participants for member checking. The duration of the interview was between 5 and 40 minutes. We considered data saturation achieved when no new code emerged from the analysis of the verbatim accounts. It occurred after the twelfth interview in the first group and after the ninth interview in the second group.

**Table 1:** Topic guide for the interviews in both sampled groups of family physicians (FPs).

**Aim**	To explore how FPS come to suspect pulmonary embolism using two groups: FPs who had recently diagnosed a case of PE; and FPs chosen using a purposeful sampling method	
**Ice breaking question**	For the first group	You have recently seen in consultation Mr/Mrs X for a suspected PE, would you tell me what happened?
	For the second group	Would you tell me about one case of pulmonary embolism you have diagnosed?
**Questions for taking the discussion further**	In your opinion, what are the risk factors for pulmonary embolism?	
	What kind of diagnostic test do you use? (ECG, saturation, d dimer, gasometry, x ray)	
	What use do you make of clinical scoring systems?	
**Reopening questions**	For the first group	How do you generally diagnose PE?
	For the second group	Some of you talked about using conviction and belief in the diagnosis of pulmonary embolism. What do you think about this idea?
		What are you looking for in particular during auscultation?
		What importance do you attach to anxiety?
		How well did you know the patient? How important was that to you?

### Data analysis

A thematic analysis was conducted using the technique of constant comparison, originating from grounded theory [16]. The first stage: open coding was done by two researchers (AC and NM) working independently without any framework for the written data. After the first stage, they shared their results. Any discrepancies between the two researchers were discussed with a third member of the research team (MB) until a consensus was reached. Through an iterative process of constant comparison, an axial coding framework was developed at the second stage. The axial coding involved linking categories found within the open coding. The same procedure of working independently before sharing the results was applied. The codebook was revised, with the 3 researchers going back to the data until mutual consent was reached. QSR N vivo 10.0 Software was used to perform the analysis.

## Results

### Sample

#### Participants

Characteristics of the participants and practices are summarized in table 2. One of the FPs interviewed had been a mentor to one of the trainees during the training period.

**Table 2:** Characteristics of the 28 FPs interviewed for data collection.

	Range	Group 1^I^	Group 2^II^
**Age of GPs**	30–65	36–63	30–65
**Male/Female**	16/12	8/5	7/7
**Number (n)**			
**Urban practice**	19	10	9
**Rural practice**	9	4	5
**Teacher or tutor**	3	1	2
**Particular interest (sports medicine)**	1	1	0
**Locum**	1	1	0

i : FPs of the patients hospitalized with PE.

ii : FPs recruited using a purposeful sampling method.

#### Case characteristics

10 FPs in the first group were correct in their suspicion and referred their patient to the emergency unit for PE. The 4 other FPs referred their patient for other reasons without having any suspicion of PE but thought about pericarditis, infection in a COPD context, pneumonia and coronary heart disease. In the second group, one FP had not even suspected a PE.

### Analysis

Analysis of the text fragments resulted in 65 open codes, which after an inductive interpretation and categorization process could be structured in 16 axial codes and 3 main categories (table 3).

**Table 3:** Themes and axial codes.

Themes	Axial codes
A polymorphic semiological picture	Many different clinical pictures
	Different way to interpret the feeling of the patient
	Uneasy diagnosis
	Contextual risk factors
	Patient's risk factors known by the FPs
	Emergency context
	Treatment
Tools used to help decision-making: ECG and the D-Dimer test	Tests: ECG and D-dimer
	Core competencies of family practice
	Scores
	Primary health care organization
The seeds of suspicion	Unusual consultation conditions
	Feelings verbalized by FPs
	Reflection on their diagnosis
	Experience of traumatic case
	Misdiagnosis or delay in diagnosis

### Key points

#### A polymorphic clinical picture

Clinical signs, which allowed FPs to form their suspicions, were so varied as to be hardly recognizable, according to the FPs interviewed. Chest pain in a PE context could occur both during effort, as well as during deep inspiration and could even be reproduced by physical palpation. The location, intensity and duration also varied without any specificity. The dyspnea was described with variable intensity from a one-off incident to almost intolerable tachypnea. Tachycardia was considered to be a helpful sign. Combined dyspnea and thoracic pain with tachycardia was directly associated with a PE diagnosis.

*“As for the symptomatology, it is very diverse. Personally, I saw many… some punctiform pains, some which appeared to be muscular, others which increased on palpation… It looked like nothing!”* (P17, female FP, rural practice, 31, group 2)

*“He was gasping like a stranded fish!”* (P10, male FP, rural practice, 57, group 1)

*“She was a bit breathless”* (P18, female FP, urban practice, 51, group 2)

*“I was very surprised because the heartbeat was very fast”* (P27, male FP, urban practice, 57, group 2)

The association between dyspnea, thoracic pain and symptoms of thrombosis facilitated the identification of PE.

*“If the thrombosis had not been there, I think it might have passed unnoticed because it was completely atypical.”* (P23, female FP, urban practice, 31, group 2)

*“She told me she had been breathless for 10 to 15 days, so I noted 10 to 15 days and she had been suffering with pain in her right calf since the previous weekend.”* (P14, male FP, urban practice, 50, group 1)

The patient's anxiety was viewed differently by different FPs. The anxiety was integral to the global picture for the FPs who had recently established a positive diagnosis. They considered it a strong clue guiding them to suspect PE. For other FPs, the anxiety was related to the dyspnea and had no specific connection with the diagnosis of PE.

*“Well, yes, I did find something else… and that's anxiety. I mean, I noticed, the dyspnea really frightened the patient, compared with other dyspnea. What alarmed me was a dyspnea that was frightening for the patient, not a usual “lambda” common dyspnea. There was anxiety and stress.”* (P25, male FP, rural practice, 62, group 2)

*“When something is wrong I think they are all anxious.”* (P16, female FP, urban practice, 34, group 2)

The absence of indicative clinical signs for other diagnoses in patients with specific complaints was in itself a strong clue, likely to evoke the diagnosis of PE. Compared to coronary heart disease and pneumonia, PE was described as a pathology with very few symptoms. Four FPs did not suspect PE before referring their patient to the emergency unit. The signs and symptoms presented were interpreted in different ways. The pattern of other pathologies, such as pericarditis or coronary heart disease, came to mind. The diagnostic process, leading to suspected PE, was dependent on the possibility of eliminating other potential pathologies. For these reasons the diagnosis of PE was considered a complex issue in primary care.

*“But it's true that if we have nothing to go on, no sign of cardiac insufficiency, and we see a person who is breathless or is in pain… When there is not much to go on, I will consider it…”* (P9, female FP, rural practice, 30, group 1)

*“She described effort-related chest pain, which lasted for 2 days. No dyspnea, no tachycardia. I did an ECG which was normal, no signs of acute coronary insufficiency, no ECG deviation from normal. I had a case of clinical angina in a patient with a long history of coronary heart disease, even though the ECG showed no change. I sent her to the emergency unit for additional exploration into chest pain which was suggestive of acute coronary syndrome.”* (P13, male FP, urban practice, group 1)

“*It's complicated. The clinical picture is different from one case to another sometimes very poor or virtually non-existent. No chest pain, no shortness of breath … Sometimes just tachycardia. And then a pulmonary embolism can cause death … It is a complicated diagnosis, very complicated.*” (P13, male FP, urban practice, 59, group 1)

#### Tools used to help decision-making: ECG and the D-dimer test

The FPs were asked about the tools they used to help them in their decision making process. The electrocardiogram had an ambivalent place: for some it was important to eliminate other diagnoses such as coronary heart disease. For FPs who had already considered PE, the ECG was seen as a waste of time. Its result would not change their management plan.

*“In my case, I did an ECG which was normal. When there is shortness of breath I systematically do an ECG. In general there is no sign.”* (P25, male FP, rural practice, 62, group 2)

*“Yes yes, we have an ECG. We do the examination but it's true that I don't check for PE. If there is any doubt and if the patient is not well … I consider it a waste of time. It will be done at the hospital. The ECG is useful for other diagnoses. When you know the patient will be referred I don't waste my time”* (P16, female FP, urban practice, 34, group 2)

*“If I consider there is typical thoracic pain or a pulmonary embolism and the ECG is normal, I will override the ECG result and refer the patient.”* (P13, male FP, urban practice, 59, group 1)

The D-dimer test was considered only a minor aid in the positive diagnosis of PE for two reasons. The first is a practical consideration: in France the test cannot be done at the FP's office but only in a laboratory. The FPs found this process too time consuming. The second reason was their need for a clear answer. The result of the test was not seen as sufficiently discriminating by some physicians. The high negative predictive probability was seen as a hindrance rather than a help in the diagnostic process.

*“Yes, but in that situation, at that moment… Anyway I thought she should be hospitalized. I could have requested the D-dimer test at the laboratory, yes, I had considered that but, in view of the history of the patient, I wanted to be certain.”* (P24, female FP, urban practice, 60, group 2)

*“It is not very reliable. It has a negative predictive value, you know. This is often more annoying than anything else…»* (P13, male FP, urban practice, 59, group)

All the FPs interviewed stated they did not use the PE prediction rules. Most of the FPs did not know of any rules for this pathology. Younger FPs were familiar with it but did not use it in daily practice. Older FPs overreacted when the scores were mentioned because many were not familiar with them. They reacted as though they were being judged just because the interviewer had posed the question. All the FPs considered it an unsuitable tool in primary care. A scoring system was treated as a way of quantifying the severity of the condition in an emergency unit. The FPs who did consider the scoring system only did so because they already believed the patient needed to be referred to the hospital. The result of the score would have had no influence on their decision-making process in the office. The FPs felt the scoring system was impersonal and at odds with a patient-centered approach and a good relationship with their patient. They insisted on the global view they had of the situation based on their examination and knowledge of the patient. The figure given by the score was seen as disconnected from the actual patient.

*“Interviewer: and what about you? Do you use other things to diagnose PE, rules for instance?*

*FP: no, no. The rule… you mean the scale… I don't bother with that.*

*I: Do you think they are useful in family practice?*

*FP: no, that's for hospital physicians, they have time to classify patients.”* (P7, male FP, rural practice, 57, group 1)

*“We are working alone; we finished our studies a long time ago. Well, of course, we don't say so but we do our examination, we look for a thrombosis, look at their previous history. We don't call it “Wells or Geneva” but we do have our clinical examination procedure.”* (P8, female FP, urban practice, 48, group 1)

*“I don't normally use them, but my examination and my knowledge of the patient ticks a lot of the boxes. I can build a global picture.” (*P22, female FP, urban practice, 37, group 2)

Some factors were highlighted by FPs working in rural areas. While FPs in urban areas could easily send the patient to the lab for a D-dimer test, where there was any doubt, rural FPs had to weigh the pros and cons carefully before referring to a specialist. They considered context to be particularly significant. They said that the distance their patient would have to travel to the hospital influenced their decision.

*“We have nothing […] And there are only two doctors in the area who can do Doppler tests. It is not that simple. You have to refer to the hospital. Here there is nothing, no lab. They have to go to the lab or the nurse has to come to their home. If we ask for the D-dimer in the morning, we will not have the answer until 3 pm. If we are in any doubt, we have to refer to the hospital.”* (P26, male FP, rural practice, 65, group 2).

*“and, in an emergency context, you just have to call and you will have a scintigraphy or a CT angiogram, you have it very quickly. The lab test, it is done in one hour in the lab next door”* (P3, male FP, urban practice, 61, group 1)

#### The seeds of suspicion

FPs told us of traumatic cases they had experienced, which paralleled the case they were discussing in the interview. A misdiagnosis with fatal consequences and a delay in diagnosis, which proved damaging for the patient, were described. These traumatic experiences reinforced the anxiety surrounding the pathology. They built a frightening picture of PE in the FP's mind. Some FPs even said that their approach to patient care was more conditioned by this traumatic experience than by objective elements.

*“Well, she had a vicious bout of influenza, she was lying in bed and I didn't prescribe any anticoagulant. It was many years ago, at that time it was not codified as it is now. I feel guilty enough admitting it, but at least, I will step up to the plate. That's the story and ever since this case I think I have been traumatized by pulmonary embolism! It always crosses my mind.”*(P8, female FP, urban practice, 48, group 1)

*“Yes… In my case, I had a patient who died at the beginning when I was covering for an FP… Now as soon as I think there might be venous disease, I follow it up.” (*P18, female FP, urban practice, 51, group 2)

*“I saw a case of thrombosis during pregnancy, actually, it was not my patient but my brother's niece and she died at the end of her pregnancy of a pulmonary embolism. It was a dramatic situation.”* (P26, male FP, rural practice, 65, group 2)

A change in a patient's attitude or behavior pattern led one FP to the diagnosis. A patient usually seen at the office asked for a home visit in an emergency and that alerted the FP. In general, FPs know their patients very well, which allows them to detect a change in the patient's condition. These changes noted in the context of a consultation, along with a sudden change in the patient's condition, were sometimes the only symptoms driving the FP towards further investigations.

*“Yes during home visits, each time, with people who usually came to the office”* (P18, female FP, urban practice, 51, group 2)

*“With a man I know well, who never complains. So I know that when he calls, it's something important.”* (P24, female FP, urban practice, 60, group 1)

*“And I know my patients very well, I have been here for 40 years and I can tell when they look ill! Yes I saw his face, and I knew that he was in trouble”* (P26, male FP, rural practice, 65, group 2)

The FPs talked about the use of their perception in diagnosis: they sensed when something was wrong, although they were unable to underpin this feeling with objective arguments. The perception of a serious prognosis decided where the patient would be sent for treatment. The FPs needed further investigation because of the sense of alarm they experienced. This feeling was described in different ways: having a “nose”, “a sense”, “an intuition”. Eighteen FPs from both sampling groups told us about this feeling. It was unrelated to the FPs' gender, age, experience or the location of their practices.

*“We sense the situation… Even if not all the signs are present… We feel things, but I can't explain them.” (*P5, female FP, 34, locum, group 1)

«*There is a notion of intensity, of underlying seriousness but you don't really know what's going on. It's true that we do think about some things in that way […] I don't know how to explain that. In some cases we said it was hard… and fortunately, we did further investigation. Yeah we did the right thing then … we started something a little faster than usual and it was beneficial.”* (P25, male FP, rural practice, 62, group 2)

*«When it comes into your head, when you're convinced, I sometimes call it “having a nose” it is true that sometimes you don't know why and you say to yourself “that's it” and you go with it. Then you have to follow it up.”* (P28, male FP, urban practice, 39, group 2)

*«Yes clinical signs and sometimes we have the… we have the “feeling”, we feel… we feel the… with converging lines of evidence, we feel that something does not fit”.* (P19, female FP, urban practice, 37, group 2)

*«Well, sometimes we found pains which looked rather dubious… We used our noses and when you know someone well, you can tell when he is not his usual self.”* (P26, male FP, rural practice, 65, group 2)

## Discussion

### Main results

The suspicion of pulmonary embolism arose out of four considerations: the absence of indicative clinical signs for diagnoses other than PE, a sudden change in the condition of the patient, a gut feeling that something was wrong and an FP's experience of previously failing to diagnose PE. The FPs interviewed did not use rules in their diagnostic process.

### Strengths and limitations of the study

As far as we know, this is the first study describing FPs' diagnostic reasoning processes in cases of pulmonary embolism. The FPs interviewed revealed their diagnostic errors, and sometimes recounted dramatic stories of failure to diagnose PE. This openness during the interview confirmed that the ambiance created by the interviewers was appropriate. The data were based on real life experiences and not on general opinions.

The first group of FPs interviewed was composed of FPs who had had a positive diagnosis of PE. We did not interview FPs who had missed a PE. In accordance with the objective of the study, we focused on the diagnostic reasoning process in cases where PE was suspected. Some FPs did not think about a PE, but about another serious disease which needed emergency care. In the second group, one FP had never even suspected a PE. We decided to include these interviews to broaden the analysis.

Another limitation of this study is the recall bias related to the FPs recruited in the second stage. We did not present a specific case to start with and sometimes their stories had occurred months or years before. In the first group the FPs did remember the cases very well which made it much easier for them to describe their diagnostic reasoning.

Two young family practice trainees carried out the interviews, which could have influenced the content of what FPs talked about. The FPs interviewed knew the study was conducted by young FPs wanting to know what occurred in primary care, rather an intern or pulmonologist who might be judgmental. However, a more hierarchical, rather than collaborative, relationship might have developed between the young interviewers and the experienced physicians. This situation may have influenced the answers of one FP who had previously been the tutor of one of the interviewers. This factor might also explain some reactions to the question about rules.

The sample was selected using two recruitment procedures. The purposive sample allowed us to interview rural FPs, female FPs, and younger FPs (see table 2). It reinforced the maximal variation of the sample.

None of the FPs interviewed used either prediction rules or the point of care D-dimer test although it is the recommended strategy according to international guidelines. Our aim was to describe what was done in real French practices, and not in an ideal situation. Relying on gut feelings in the first stage of the diagnostic process does not exclude the usefulness of clinical decision rules such as the Well's rule. On the contrary, gut feelings should trigger the next clinical process and especially the use of rules such as the Well's rule. The fact that the FPs interviewed revealed their real practice which, although far from what is recommended, is nevertheless another illustration of the openness achieved during the interview.

### Key points

The diversity of the clinical pictures of PE did not provide a foundation on which FPs could base their decisions. FPs interviewed in this study, attached importance to the presence of symptoms of thrombosis and patient anxiety. They mainly emphasized the absence of objective and indicative signs in parallel with patients' complaints. A sudden change in the condition of the patient was considered as the most important indication. This point had already been raised, in cases where coronary heart disease was suspected [17], as a reason for referral of patients with chest pain [18], and in cases of meningococcal infection in children [19]. Background knowledge about the patient and person-specific discrepancy were tools used where a serious condition was suspected. The results of our study contribute to the improvement of a specific strategy in primary care: knowing the patient, his risk factors and being sensitive to a discrepancy in the patient's behavior seemed to be decisive for FPs when clinical signs were vague but serious conditions were suspected.

The FPs also based their decision on what they felt during the consultation. That uneasy feeling experienced by the FPs interviewed matched the sense of alarm described in the “gut feeling” concept [20]. This sense of alarm implies that an FP worries “about a patient's health status, even though he/she has found no specific indications yet”; it is a sense that ‘there's something wrong here’” [20]. Their sense of alarm involves specific management whereby the patient has to be referred to an emergency unit, or to a specialist to prevent serious health problems. The gut feeling concept was originally formalized from statements raised in family medicine in the Netherlands and in Belgium [21]. Its transculturality was proven in a Romance language [22]. The FPs interviewed in our study affirmed the existence of the sense of alarm in the specific situation of a suspected PE. Van Den Bruel et al. found that a gut feeling had a higher specificity than a clinical impression in the context of serious infection in children in primary care. The authors recommended that this gut feeling should not be ignored in diagnostic reasoning but has to be used as a red warning flag [23]. One factor which contributed to gut feeling was the perception of parental concern. In our study FPs who had recently diagnosed PE, attached importance to the anxiety expressed by the patient. They felt that this anxiety was indicative of PE whereas other FPs considered the anxiety to be the result of the dyspnea. In our study the patient's anxiety and the sense of alarm perceived by the FPs steered the diagnostic reasoning process.

The FPs interviewed did not use the Wells rule during their diagnostic process because this prediction rule was not considered a helpful tool for detecting PE in primary care. The Wells score, combined with a qualitative D-dimer test, safely and efficiently excludes PE in primary care [11]. This procedure provides a concrete way to deal with the suspicion of PE depending on whether the probability is high or low: if the score is ≤4, the probability of PE is low and a D-dimer test is required and if the score is >4, the probability of PE is moderate to high and further investigations in hospital (compression ultrasonography, pulmonary vascular imaging) are required [24]. The Wells rule brings a synthesis between clinical symptoms, clinical signs and the physician's assessment with the allocation of three points to the physician's clinical judgement of whether PE is more likely than an alternative diagnosis. The physician may express his feeling of alarm with these three points. This first stage in the suspicion of PE, including the sense of alarm, should drive the FPs forward to the second stage of the diagnostic process using the Well's prediction rule. This strategy has a clinical impact in the FP's decision and fits the criteria of a relevant rule for decision-making in primary care [25]. How can we explain this non-use of a safe decision strategy by the FPs interviewed in this study? The use of the point of care D-dimer test is not widespread in France: all the FPs interviewed had to refer their patients to an independent laboratory to have the blood sample analysed. Most of the older FPs had never heard of the Wells rule; indeed, even if the younger ones had learnt how to use it during initial training at the university, they had not used the scores in the cases they related. All the FPs interviewed agreed about their willingness to use their global evaluation of the case, guided by their knowledge of the patient. For them, using a score did not fit the patient-centered approach [26]. This is consistent with one of the main aspects of the definition of family practice where the patient-centered approach belongs to the core competencies of the discipline [5]. Clinical impression, global empirical clinical assessment (also called “gestalt”) are the tools used to qualify a holistic rather than an atomistic approach based on clinical context [27]–[29]. Lucassen et al. compared the predictive value of the gestalt and clinical decision rule when used in combination with D-dimer testing for excluding pulmonary embolism [10]. The sensitivity of gestalt was similar to clinical rules (Wells, Geneva, Revised Geneva) but its specificity was lower [10]. Using a point of care D-dimer test combined with the Wells rule was useful in reducing false positives [10]. The FPs interviewed apparently gave priority to sensitivity rather than specificity. This may be explained by the fact that, in the context of low incidence serious diseases [30], the FPs appear to attach greater value to true-positive decisions (correct decisions to provide care to patients who need it) than to true-negative decisions (correct decisions to withhold care from patients who do not need it). Providing care for patients whom they suspect may be suffering from a life threatening disease, is possibly considered much more important than withholding care from a patient in good health [25].

Moreover the FPs interviewed told us about diagnostic errors they or their colleagues had made when diagnosing PE and how the sense of alarm popped up from their experience and knowledge. This description of the sense of alarm fits into the Reason's model of error prevention in decision making [31]. The experienced FPs function as expert decision makers: as a result of their experience, they have established a set of spontaneous reactions to patterns of diseases they identify at a glance [32]. These rapid, effortless and unconscious ways of thinking are named heuristics: they are considered as powerful tools [33] but with specific errors [34]. When the FPs are confronted by a new situation they have to call upon slow and demanding analytical reasoning. The “uneasy feeling” perceived by the FP corresponded to a perception of cognitive dissonance: the mismatch situation with pre-established patterns triggered the sense of alarm in the FP [32]. The perception of alarm compelled the physician to quit his routine-based reasoning to an analytical reasoning by generating and considering the PE hypothesis [35], [36]. The sense of alarm acted as a feedback mechanism, allowing the questioning of a possibly wrong decision at a very early stage of the diagnostic process. This feeling is based on medical, experiential and contextual knowledge. The traumatic experiences FPs narrated during the interviews are images which are tagged with negative affect in their memories [37]. This shortcut was named the affect heuristic [38]. In the situation where PE was suspected, we may hypothesize that this affect heuristic emphasized the FP's sense of alarm, forcing him to switch to analytical reasoning. The sense of alarm was used here as a tool to prevent the diagnostic error of missing a PE.

### Implications for practice and future research

The accuracy of the sense of alarm needs to be evaluated in the context of PE. The data were collected in a qualitative retrospective manner. We cannot generalize from our results without testing them in a prospective way on a large sample of situations in primary care. A short questionnaire has been developed to determine gut feelings in primary care settings [39]. We are aiming to use this questionnaire to study the predictive value of the sense of alarm when confronted with dyspnea and/or thoracic pain at the office. The objective is to compare the results to the questionnaire, filled in at the end of the consultation, with the diagnostic outcome four weeks later. Before implementing the gut feelings concept in educational programs, we need to study its accuracy in real settings.

## Conclusions

This study aimed to describe an early stage in the diagnostic process of FPs who suspected pulmonary embolism at their office. The absence of indicative clinical signs for diagnoses other than PE, a sudden change in the condition of the patient, and the FP's experience of previously failing to diagnose PE, as well as a sense of alarm were the main determinants of the decision to refer. A decision rule was not used at all. The sense of alarm was used as a tool to prevent the diagnostic error of missing a PE. The diagnostic accuracy of this aspect of gut feelings has to be evaluated before being recommended or taught.
